# SIRPγ limits effector differentiation of human CD8 T cells in response to subthreshold TCR-signaling

**DOI:** 10.1093/immhor/vlag027

**Published:** 2026-06-21

**Authors:** Megan Morse, Xanthie Rodriguez, Erika Delarosa, Sierra Rodriguez, Juma Shanil, Sushmita Sinha

**Affiliations:** Department of Biology, Texas Woman’s University, Denton, TX, United States; Department of Biology, Texas Woman’s University, Denton, TX, United States; Department of Biology, Texas Woman’s University, Denton, TX, United States; Department of Biology, Texas Woman’s University, Denton, TX, United States; Department of Nutrition Sciences, Texas Woman’s University, Denton, TX, United States; Department of Biology, Texas Woman’s University, Denton, TX, United States

**Keywords:** autoimmunity, human T cells, differentiation, inflammation, SIRPγ

## Abstract

Signal regulatory protein gamma (SIRPγ) is a T cell–specific surface receptor in the human immune system with previously undefined function in human CD8 T cell differentiation. We report that SIRPγ expression varies substantially across individuals and stratifies CD8 T cell differentiation states. Individuals with low SIRPγ expression exhibit an increased frequency of CD27^−^CD45RO^+^ effector-like and CD27^−^CD45RO^−^ terminally differentiated CD8 T cells, while high expressors retain a predominance of naïve and central memory cells. To investigate the functional role of SIRPγ, we performed small interfering RNA–mediated knockdown in naïve human CD8 T cells. Under suboptimal TCR stimulation, *SIRPG* knockdown drove robust effector-like differentiation marked by increased CD45RO expression, T-bet upregulation, and enhanced production of TNF-α, IFN-γ, and granzyme B. This phenotype was not recapitulated by CD47 blockade, indicating that SIRPγ modulates differentiation through a CD47-independent mechanism. These findings identify SIRPγ as a negative regulator of CD8 T cell effector programming under limiting stimulatory conditions. Interindividual variability in SIRPγ expression may influence immune homeostasis and susceptibility to immunopathology, highlighting SIRPγ as a potential therapeutic target in settings of dysregulated T cell responses.

## Introduction

During the COVID-19 pandemic, we have seen the interindividual variability in immune responses to SARS-CoV-2. This variability highlights a key long-standing and unresolved question in immunology: why do some individuals mount a balanced immune response that eliminates infection with no harm to host cells, whereas others have exaggerated immune responses that can cause significant tissue injury and may precipitate autoimmunity?^1–9^ Despite extensive research, the host-intrinsic factors that fine-tune T cell responses and immune homeostasis remain incompletely understood. Uncovering these mechanisms is critical for addressing not only infection, but also autoimmunity and cancer, in which immune balance is often disrupted.

T cells play a critical role in shaping a balanced immune response to foreign antigens and self-antigens. They do so by directly recognizing molecules expressed on the cell surface and secreting factors that drive or dampen local inflammatory responses. One such molecule is signal regulatory protein gamma (SIRPγ), an immunomodulatory protein that is uniquely expressed on the cell surface of human T cells.^10^^,^^11^ Variants in the *SIRPG* gene have been associated with type 1 diabetes,^12–15^ relapsing-remitting multiple sclerosis, and maintaining long-term vaccine responses.^16^ We found that humans express varying levels of SIRPγ on their T cells, and that T cells with less SIRPγ surface expression exhibit heightened effector status.^17^ Importantly, our prior studies showed that SIRPγ^low^ T cells are significantly enhanced in individuals with autoimmune diseases such as type 1 diabetes and relapsing-remitting multiple sclerosis.^18^ This suggests a potential role for SIRPγ as a regulatory checkpoint whose dysfunction contributes to human autoimmunity. Since the publication of our original findings, a slew of papers has emerged describing the role of SIRPγ in chronic immune responses,^19^ lung squamous cell carcinoma,^20^ cancer stem-like cells,^21^ and type 1 diabetes.^15^^,^^22^ However, its specific role in the immune system, particularly naïve CD8 T cells, remains unidentified.

Naïve CD8 T cells, upon encountering antigen, undergo extensive phenotypic and functional changes, transitioning through central memory (CM), effector memory (EM), and terminally differentiated effector (TEMRA) stages, and must be tightly regulated to prevent inappropriate effector commitment, especially under low-affinity or suboptimal stimulation. Previous studies, including our own, have shown that *SIRPG* expression is elevated in naïve and CM CD8 T cells, with expression progressively declining as cells transition to EM and TEMRA states.^17^^,^^19^ While this trend suggests a link between SIRPγ and effector stages, it has not been clear whether SIRPγ plays an active role in modulating differentiation or is simply a marker of T cell maturity.

In this study, we address this knowledge gap by investigating the functional role of SIRPγ in regulating human CD8 T cell differentiation. We demonstrate that interindividual variability in SIRPγ expression—both naturally occurring and experimentally induced via small interfering RNA (siRNA)—profoundly influences the balance between naïve and effector T cell subsets. Specifically, *SIRPG* knockdown (KD) in naïve human CD8 T cells leads to premature effector-like differentiation under suboptimal TCR stimulation, characterized by CD27^low^ CD45RO^+^ phenotype and elevated expression of effector cytokines and transcription factors. Notably, these effects were independent of CD47, the known SIRPγ ligand, suggesting a distinct signaling role for SIRPγ in regulating differentiation thresholds.

By identifying SIRPγ as a molecular brake that prevents premature effector programming, our findings reveal a mechanism by which human T cells resist inappropriate activation under weak antigenic signals. These insights are particularly relevant to contexts such as autoimmunity, chronic infections, and vaccine responsiveness, in which immune dysregulation can lead to tissue damage or immune exhaustion. Moreover, given the lack of a murine ortholog, this work underscores a uniquely human regulatory axis in T cell biology—one that may offer novel therapeutic opportunities for modulating effector responses in disease.

## Methods

### Study participants

De-identified leukoreduction buffy coat samples were collected from 44 healthy donors at Gulf Coast Regional Blood Center. All studies were conducted in compliance with the Declaration of Helsinki and approved by the Texas Woman’s University Institutional Review Board. The mean age of the healthy donors was 40 ± 18 yr, with a sex distribution of 21 males and 19 females.

### Cell preparation, KD, and activation

Peripheral blood mononuclear cells (PBMCs) were isolated from buffy coats using a density gradient with Lymphoprep (Stemcell Technologies). SIRPγ^high^ and^.^ SIRPγ^low^ donors were identified as described previously.^17^ PBMC samples from SIRPγ^high^ and^.^ SIRPγ^low^ donors were used for determining the frequencies of naïve, CM, EM, and TEMRA cells in SIRPγ^high^ vs^.^ SIRPγ^low^ donors. Naïve CD8 T cells were then purified from the PBMC samples from SIRPγ^high^ donors using a CD8 Naïve T Cell Sorting Kit from Stemcell Technologies, following the manufacturer’s instructions. The purity of the naïve CD8 T cells was consistently >99%. The purified cells were cultured with either scrambled control siRNA or *SIRPG* siRNA (Horizon Discovery), with KD performed according to the manufacturer’s guidelines. For activation, cells were treated with suboptimal concentrations of anti-CD3 (Tonbo Biosciences) for 48 h, as described previously. We chose suboptimal activation of naïve CD8 T cells because our previous research demonstrated that SIRPγ^low^ CD8 T cells have lower activation thresholds.^17^ Following incubation, the plates were centrifuged and the cells were used for flow cytometry staining.

### Flow cytometry antibody staining

Fluorescently conjugated antibodies used in the study were purchased from BioLegend or Tonbo Biosciences. Cell samples were washed with phosphate-buffered saline (PBS) (Mediatech Cellgro) and then stained with the appropriate fluorescently labeled antibodies. After staining, cells were resuspended in 1% paraformaldehyde (Tonbo Biosciences). Flow cytometry data were acquired using a CytoFLEX Flow Cytometer (Beckman Coulter) and analyzed with FlowJo software (BD Biosciences). Intracellular flow cytometry staining was performed to detect IFNγ, TNFα, granzyme B, and T-bet. Briefly, cells were fixed and permeabilized (Tonbo Biosciences) before intracellular staining.

### CD47 neutralization assay

Sorted naïve CD8 T cells were plated in 96-well plates, and control-IgG or anti-CD47 neutralizing antibody was added.^23^ Cells were activated after 2 h with suboptimal concentrations of anti-CD3. Following incubation, the plates were centrifuged and the cells were used for flow cytometry staining.

### Statistical analysis

Data were compared using unpaired or paired *t* test (as indicated in each figure legend), and *P* < 0.05 was considered significant. Data in Fig. S1 were compared using 2-way analysis of variance with Tukey’s post hoc analysis, and *P* < 0.05 was considered significant.

## Results

### SIRPγ expression varies between individuals and stratifies CD8^+^ T cell differentiation states

**Figure 1:**
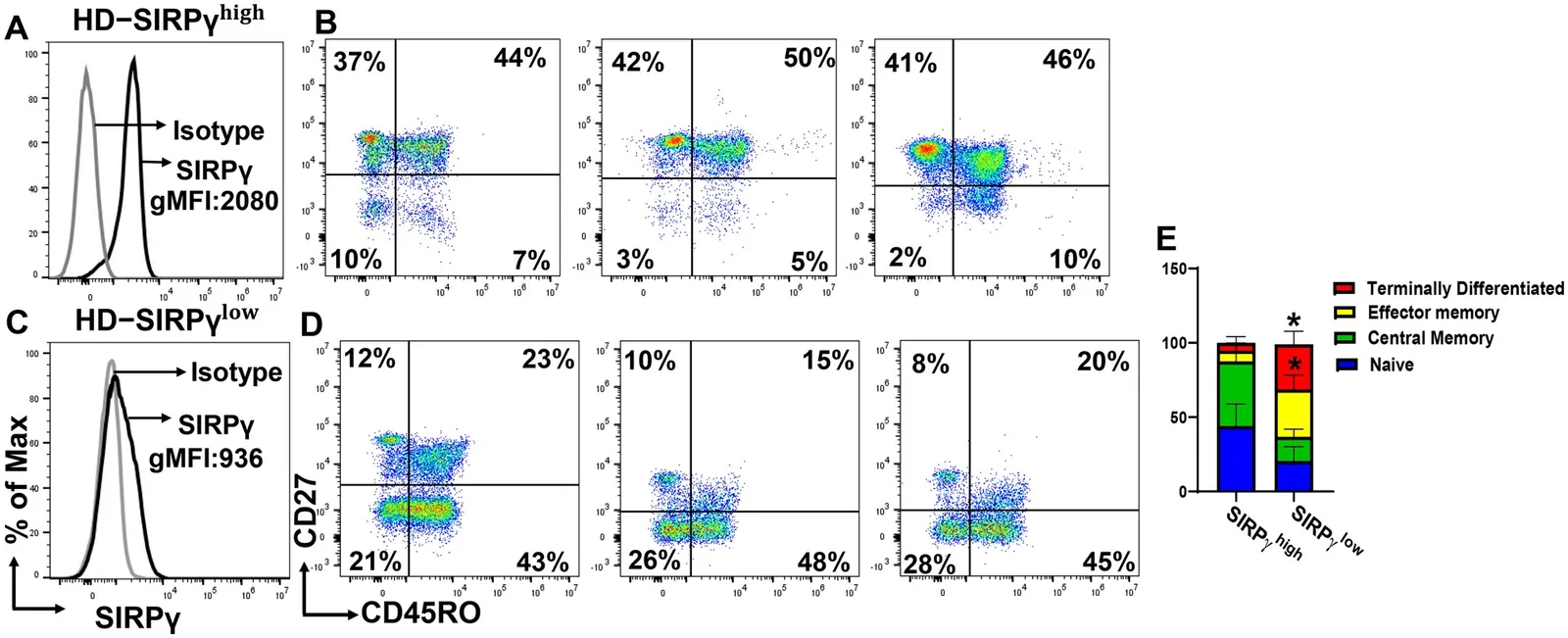
SIRPγ^low^ carriers have significantly increased frequencies of effectors and terminal effectors in peripheral blood. PBMCs were isolated from 50 healthy donors (HDs) and stained with fluorescently conjugated antibodies. SIRPγ^high^ versus SIRPγ^low^ carriers were identified based on a previously described strategy. Representative histograms showing SIRPγ expression on bulk CD8 T cells from a SIRPγ^high^ and a SIRPγ^low^ donor are shown in panels A and C, respectively. Dot plots showing the distribution of CD8 T cell subsets from 3 independent SIRPγ^high^ and SIRPγ^low^ donors are shown in panels B and D, respectively. (E) Cumulative data show that SIRPγ^low^ individuals exhibit a significantly higher frequency of effector-like (CD45RO^+^CD27^−^) and terminally differentiated (CD45RO^−^CD27^−^) CD8 T cells compared with SIRPγ^high^ donors. SIRPγ^high^ individuals predominantly retained naïve and CM-like populations, suggesting a more balanced differentiation state. Statistical analysis was done using an unpaired *t* test. *P < 0.001. gMFI, geometric mean fluorescence intensity.

We have shown that SIRPγ expression on CD8 T cells varies significantly among individuals.^17^ In Fig. 1, SIRPγ^low^ and SIRPγ^high^ donors were identified using two complementary approaches, as described previously: (i) genotyping for the rs2281808 SNP and (ii) flow cytometric assessment of SIRPγ surface expression on CD8 T cells. In the current study, CD8 T cells from 50 unique donors were analyzed. Of these, 4 individuals were homozygous carriers of the rs2281808 risk allele, and all 4 exhibited markedly reduced SIRPγ surface expression on CD8 T cells (Fig. 1C), consistent with prior reports. To increase the number of SIRPγ^low^ individuals available for analysis, data from 4 additional, previously characterized donors who were homozygous for the rs2281808 risk allele and exhibited low SIRPγ expression were included. These donors were biologically independent from the current cohort and do not overlap with the 50 donors included in the present study. Beyond natural variability, SIRPγ expression is also influenced by T cell differentiation. Specifically, SIRPγ is robustly expressed on naïve and CM T cells but decreases as T cells differentiate into EM and TEMRA subsets (Fig. S1). This pattern suggests that SIRPγ may play a role in regulating T cell differentiation. To investigate this further, we compared the differentiation profiles of CD8 T cells from individuals with high and low SIRPγ expression. Our findings revealed that individuals with low SIRPγ expression have a significantly higher proportion of effector-like (CD45RO^+^CD27^−^, SIRPγ^high^ vs. SIRPγ^low^; mean 6.7 ± 3.7 vs. 32 ± 9.6; *P* < 0.00001) and terminally differentiated T cells (CD45RO^−^CD27^−^ SIRPγ^high^ vs. SIRPγ^low^; mean 4.7 ± 4 vs. 30 ± 8.7; *P* < 0.00001) (Fig. 1D, E), while those with high SIRPγ expression predominantly exhibit naïve and CM-like cells (Fig. 1B, E). These observations suggest that low SIRPγ expression may skew T cell differentiation toward more differentiated, effector-like states, potentially disrupting immune homeostasis in SIRPγ^low^ individuals. This variability provides a unique opportunity to explore how differential SIRPγ expression impacts T cell differentiation, particularly the transition between naïve, CM, EM, and TEMRA states.

### *SIRPG* KD promotes effector-like differentiation of naïve human CD8 T cells under suboptimal stimulation

**Figure 2:**
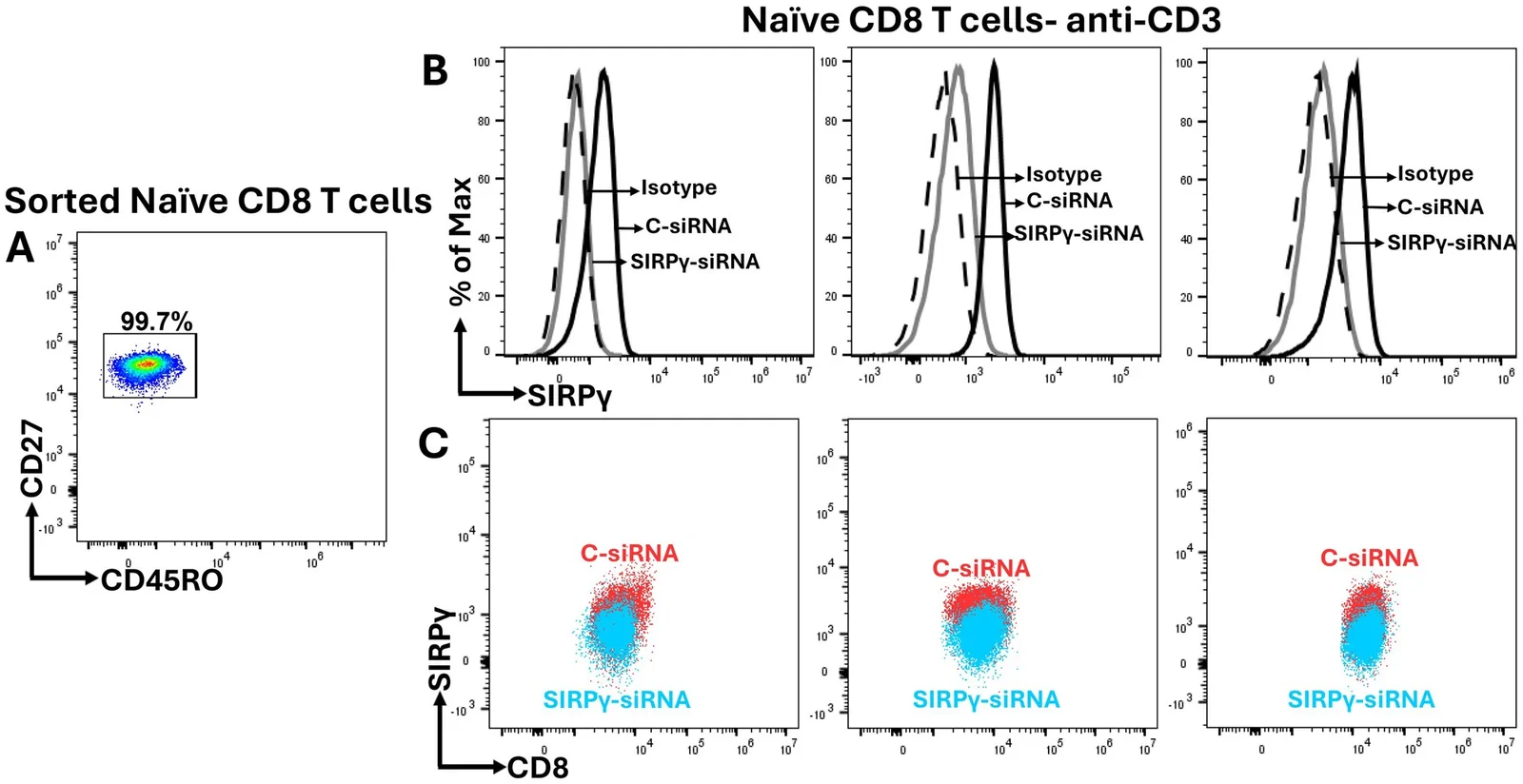
siRNA-mediated KD of SIRPγ in naïve human CD8 T cells. (A) Naïve human CD8 T cells were purified from PBMCs of SIRPγ^high^ healthy donors using a stem cell kit. Cells were transfected with either control scrambled siRNA or a SMARTpool Accell siRNA targeting *SIRPG*, following the manufacturer’s instructions. (B, C) Representative flow cytometry plots showing SIRPγ surface expression in naïve CD8 T cells 48 h post-transfection. SIRPγ KD resulted in a marked reduction in surface expression compared with the scrambled siRNA control. Isotype staining is shown in black dotted lines.

**Figure 3:**
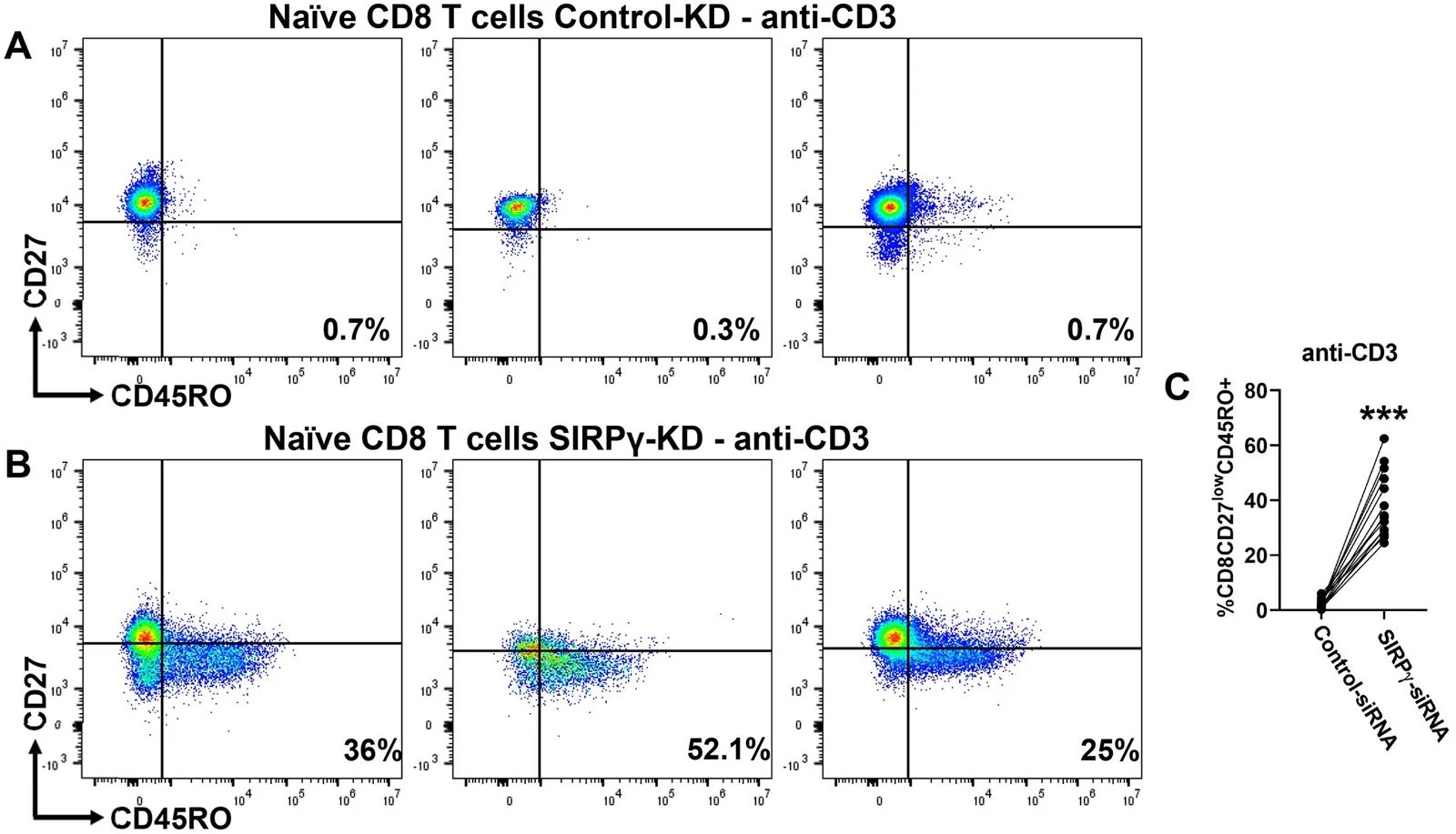
SIRPγ KD promotes effector-like differentiation of naïve human CD8 T cells under suboptimal stimulatory conditions. (A, B) Representative flow cytometry dot plots showing CD27 and CD45RO expression in naïve CD8 T cells transfected with either (A) control siRNA or (B) SIRPG-specific siRNA following suboptimal anti-CD3 stimulation. (C) Quantification of CD27^low^CD45RO^+^ CD8 T cells from 14 independent SIRPγ^high^ donors reveals a significant increase in effector-like differentiation upon SIRPG KD. (C) Statistical analysis was done using a paired *t* test. ***P < 0.001.

To determine whether *SIRPG* KD drives effector-like differentiation in naïve human CD8 T cells, we transfected purified naïve CD8 T cells with control-scrambled siRNA or siRNA targeting *SIRPG.* Cells were either left unstimulated or subjected to suboptimal stimulation with anti-CD3 antibodies for 72 h, and differentiation was analyzed by flow cytometry. As shown in Fig. 2, SIRPγ expression was efficiently reduced in siRNA-treated naïve CD8 T cells (Fig. 2A, B). Effector-like cells were defined by the surface phenotype CD27^low^CD45RO^+^. Upon stimulation with suboptimal anti-CD3, *SIRPG* KD substantially increased the proportion of CD27^low^CD45RO^+^ effector-like cells across all donors analyzed. Control samples exhibited low levels of differentiation (ranging from 0.3% to 5%; mean 2.5 ± 1.7%) (Fig. 3A, C), whereas *SIRPG*-deficient cells demonstrated a striking rise in effector-like cells, ranging from 24% to 60% (mean 38 ± 11%) (Fig. 3B, C) under identical conditions. To determine the effects of SIRPγ KD on naïve T cells under optimal TCR stimulation, we assessed CD45RO upregulation following stimulation with a high dose of anti-CD3 (1 µg/mL). Based on our prior work, 1 µg/mL anti-CD3 was used as an established condition for robust activation of naïve T cells.^17^ In contrast to suboptimal stimulation conditions, both control KD and SIRPγ KD naïve CD8 T cells upregulated CD45RO in response to strong TCR signaling (Fig. S2A, B). Although SIRPγ KD naïve CD8 T cells showed a trend toward increased CD45RO upregulation, the difference between the groups did not each significance. These results demonstrate that *SIRPG* KD markedly enhances the capacity of naïve CD8 T cells to adopt an effector-like phenotype, particularly under suboptimal TCR stimulation. This suggests that SIRPγ acts as a regulatory checkpoint, restraining premature effector differentiation when TCR signals are weak.

### Effector-like differentiation of naïve CD8 T cells following SIRPγ KD occurs independently of CD47 interaction

**Figure 4:**
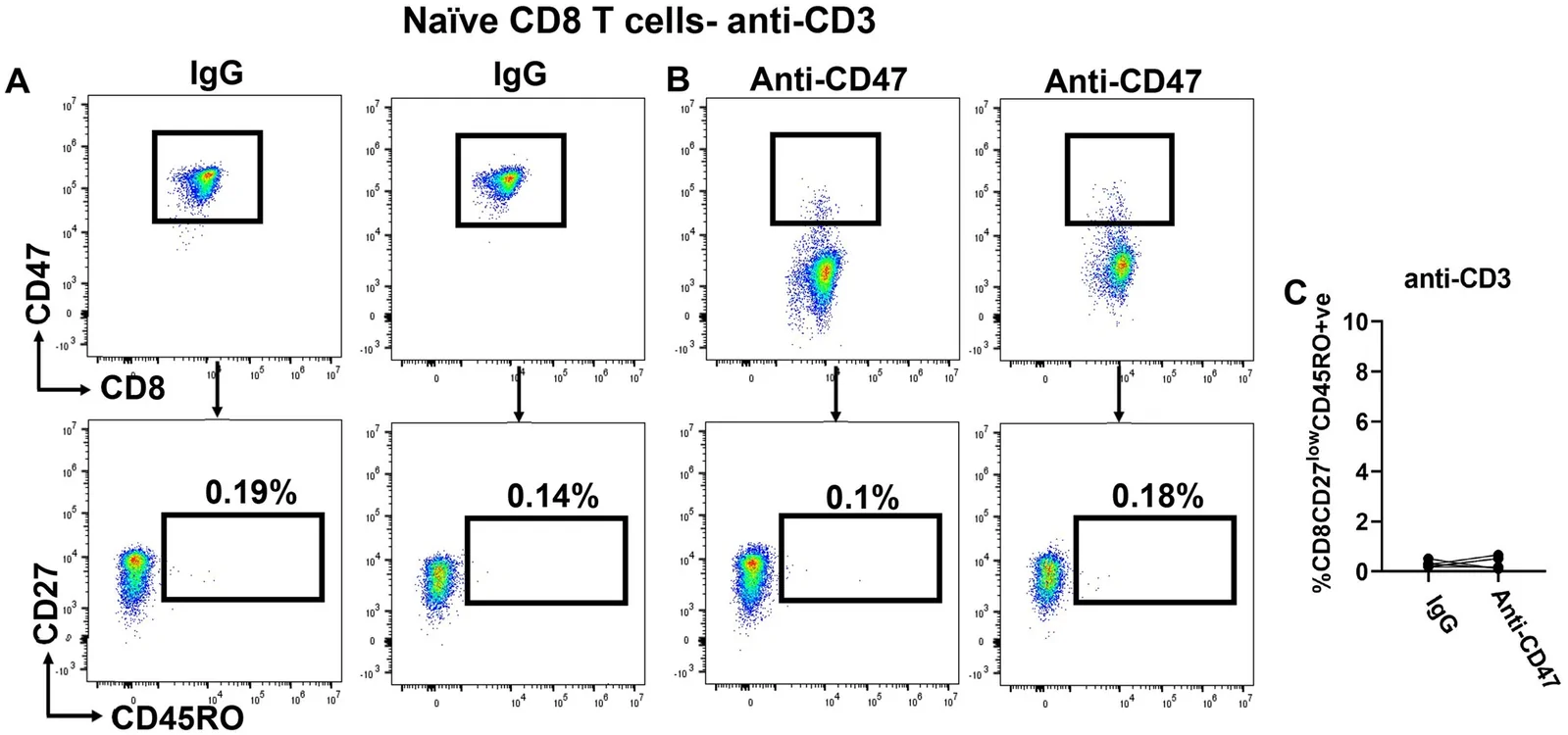
CD47 blockade does not promote effector-like differentiation of naïve CD8 T cells under suboptimal stimulation. Naïve human CD8 T cells from SIRPγ^high^ donors were pretreated with a neutralizing anti-CD47 antibody or isotype control, followed by suboptimal anti-CD3 stimulation for 72 h. (A) Flow cytometry analysis confirmed effective blockade of CD47. (B) Expression of CD45RO was assessed poststimulation. CD47-neutralized cells did not show an increased CD45RO expression compared with isotype-treated control cells. Data are representative of 6 independent donors. These results indicate that SIRPγ-mediated regulation of CD8 T cell differentiation occurs via a CD47-independent mechanism.

To determine whether the interaction between SIRPγ and its known ligand CD47 is required for effector-like differentiation of naïve CD8 T cells, CD47 was neutralized on freshly isolated naïve human CD8 T cells using a blocking antibody. Cells were then subjected to suboptimal TCR stimulation, a condition that promoted CD45RO upregulation following SIRPγ KD. Flow cytometric analysis confirmed effective blockade of CD47 on the cell surface (Fig. 4A, B). However, in contrast to the phenotype observed with SIRPγ KD (Fig. 3B), CD47-neutralized naïve CD8 T cells did not upregulate CD45RO upon suboptimal stimulation (Fig. 4B, C). Thus, removal of CD47 ligand engagement alone is insufficient to induce effector-like differentiation. These results demonstrate that the effector-like differentiation observed following SIRPγ KD is not mediated by loss of CD47 interaction, indicating that SIRPγ’s regulatory role in CD8 T cell differentiation occurs independently of its interaction with CD47. This suggests the involvement of CD47-independent signaling mechanisms downstream of SIRPγ.

#### SIRPγ KD enhances T-bet expression in emerging effector CD8 T cells

**Figure 5:**
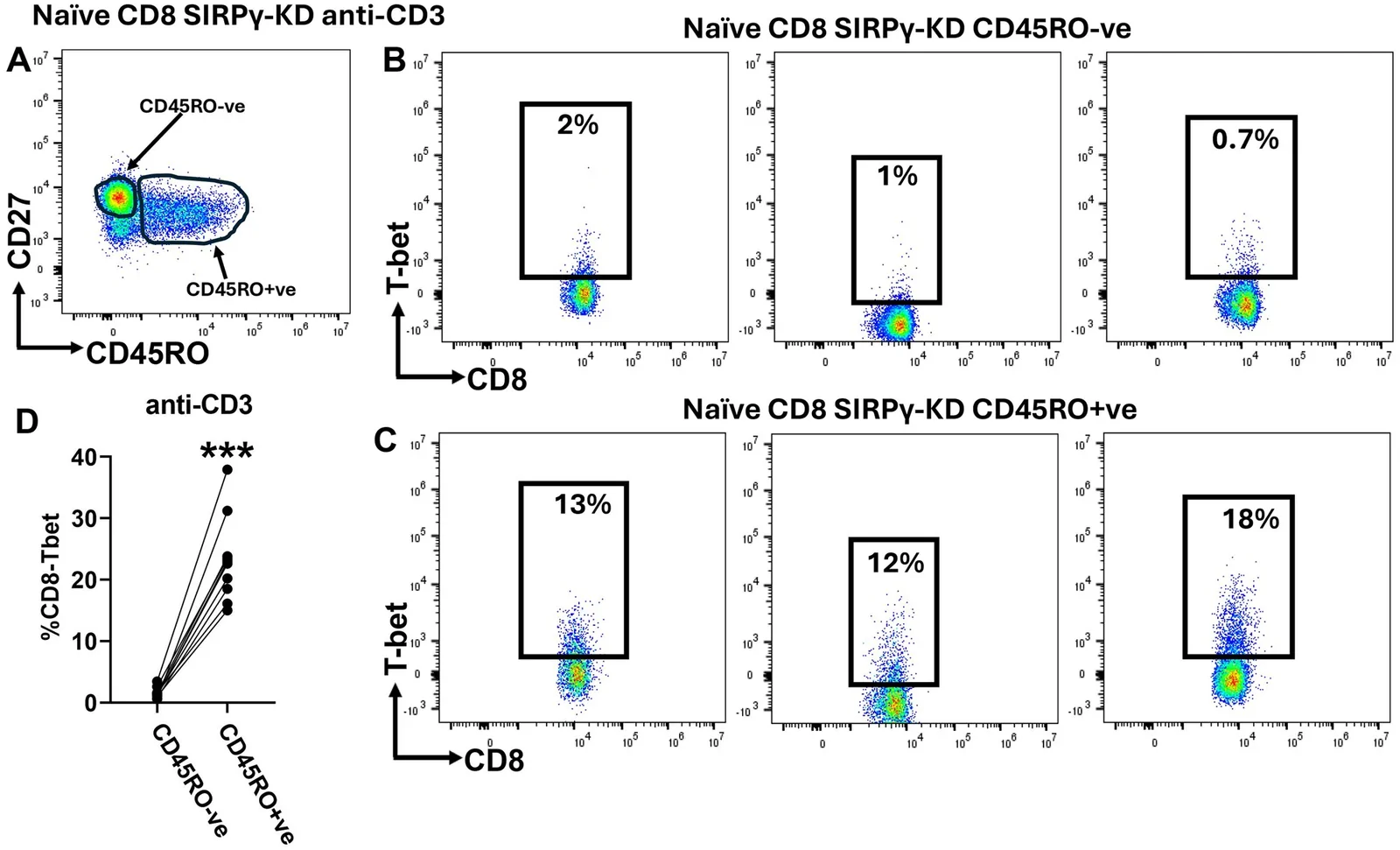
SIRPγ KD promotes T-bet expression in CD8 T cell effectors. (A) Representative flow cytometry plot showing CD45RO^−^ and CD45RO^+^ CD8 T cell subsets after SIRPγ KD. (B, C) Intracellular T-bet expression in (B) CD45RO^−^ and (C) CD45RO^+^ subsets, assessed by flow cytometry in SIRPγ KD cultures from 3 independent donors. CD45RO^+^ cells consistently exhibited significantly higher T-bet expression compared with CD45RO^−^ cells across donors. (D) Cumulative data is representative of 10 independent donors. These findings are consistent with our prior observations that T-bet expression inversely correlates with SIRPγ levels in CD8 T cells, supporting a model in which reduced SIRPγ promotes effector differentiation through T-bet–mediated transcriptional programming. Statistical analysis was done using a paired *t* test. ***P < 0.001.

We have previously shown that T-bet expression correlates inversely with SIRPγ expression in human CD8 T cells, raising the possibility that SIRPγ may restrain effector differentiation by limiting T-bet expression. To directly test this, we analyzed T-bet expression in SIRPγ KD cultures following suboptimal TCR stimulation, comparing CD45RO^+^ (effector-like) (Fig. 5A, C) and CD45RO^−^ (naïve) (Fig. 5A, B) subsets by intracellular flow cytometry. CD45RO^+^ cells showed a clear increase in T-bet expression relative to their naive counterparts in SIRPγ KD cultures (SIRPγ KD, CD45RO− vs. CD45RO+; mean 1 ± 0.8% vs. 23 ± 5%; *P* < 0.001; 127 ± 146 vs. 1576 ± 546) (Fig 4C, D). These results suggest that loss of SIRPγ facilitates T-bet upregulation specifically in CD8 T cells that are transitioning into an effector-like state, even under suboptimal stimulation.

### SIRPγ KD-induced CD45RO^+^ cells function as bona fide effectors

**Figure 6:**
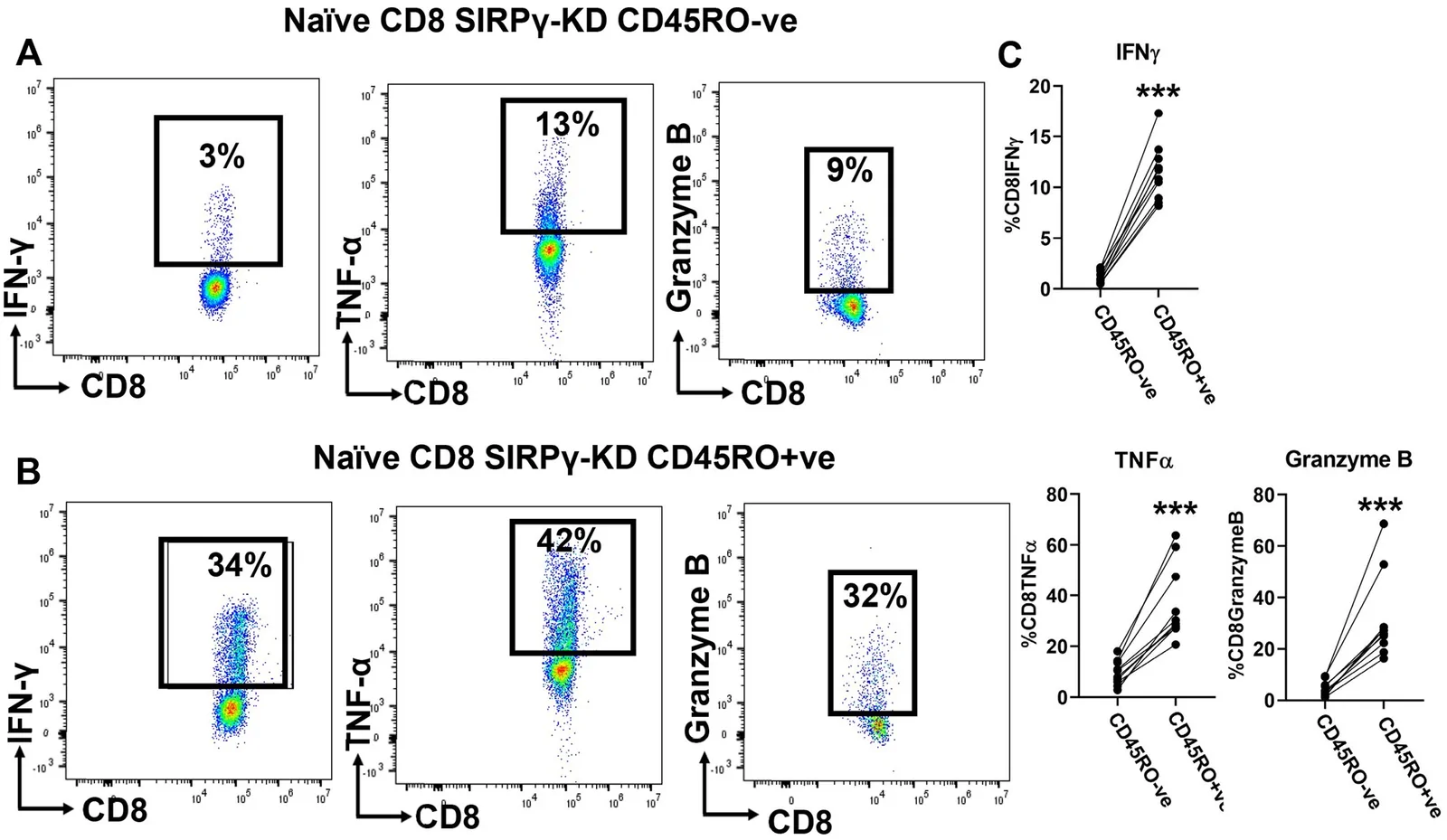
SIRPG KD enhances effector cytokines and granzyme production in CD45RO^+^ CD8 T cells. Naïve human CD8 T cells from SIRPγ^high^ donors were subjected to SIRPG KD and subsequently analyzed for CD45RO expression as shown in Fig. 4A. To evaluate effector function, intracellular levels of TNFα, IFNγ, and granzyme B were measured by flow cytometry in (A) CD45RO^−^ and (B) CD45RO^+^ subsets. CD45RO^+^ cells produced significantly higher levels of TNFα, IFNγ, and granzyme B compared with their CD45RO^−^ counterparts, indicating that SIRPG KD promotes functional differentiation of CD8 T cells toward an effector phenotype. (C) Cumulative data from 10 independent SIRPγ^high^ donors. Statistical analysis was done using a paired *t* test. ***P < 0.001.

Given the enhanced expression of T-bet in CD45RO^+^ effector-like populations upon SIRPγ KD, we next examined whether these cells functionally behave as effectors. Importantly, we tested this under suboptimal stimulation conditions, using low-dose anti-CD3 to better reveal the impact of SIRPγ loss on early activation thresholds—conditions that typically do not drive robust cytokine production in naïve T cells. Using intracellular cytokine staining and flow cytometry, we compared TNF-α, IFN-γ, and granzyme B production between CD45RO^+^ and CD45RO^−^ subsets in SIRPγ-deficient cultures after suboptimal anti-CD3 stimulation (Fig. 6A, B). As expected, CD45RO^−^ cells (representing the naive pool) showed minimal cytokine production under these conditions (Fig. 6A, C), whereas CD45RO^+^ cells consistently showed a significantly higher levels of effector molecules (Fig. 6B, C). We observed a marked increase in TNF-α production in the CD45RO^+^ population across all donors with several donors exhibiting more than a 2- to 3-fold increase (SIRPγ KD, CD45RO− vs. CD45RO+; mean 10 ± 5% vs. 33 ± 15%; *P* < 0.001) (Fig. 6B, C). A similar pattern was observed for IFN-γ, where CD45RO^+^ cells produced significantly more cytokine compared with their CD45RO^−^ counterparts (SIRPγ KD, CD45RO− vs. CD45RO+; mean 1 ± 0.6% vs. 12 ± 2.7%; *P* < 0.001) (Fig. 6B, D). In addition, we examined granzyme B expression as a surrogate for cytotoxic potential. Again, the CD45RO^+^ subset showed enhanced expression as compared with their CD45RO^−^ counterparts (SIRPγ KD, CD45RO− vs. CD45RO+; mean 5 ± 3% vs. 31 ± 16%; *P* < 0.001) (Fig. 6B, E). These results demonstrate that CD45RO^+^ cells emerging after SIRPγ KD not only are phenotypically distinct, but also functionally resemble effector cells, producing key cytokines and cytotoxic molecules. The elevated production of TNF-α, IFN-γ, and granzyme B confirms that these cells behave as bona fide effectors, supporting the idea that SIRPγ sets an activation threshold that normally limits premature or excessive effector differentiation.

## Discussion

SIRPγ is a member of the SIRP family that is selectively expressed on human T cells in the immune system.^11^ While its expression patterns across CD8 T cell subsets have been described previously by us and others, its functional role in regulating human CD8 T cell differentiation has remained poorly understood. Here, we provide new mechanistic and functional insights into how SIRPγ constrains effector differentiation and shapes CD8 T cell fate decisions, particularly under suboptimal stimulatory conditions.

Consistent with our prior observations and those reported by others, we confirm that SIRPγ expression is highest on naïve and central memory CD8 T cells and declines with progressive differentiation into effector and terminally differentiated states. Extending these findings, our current work reveals that this expression pattern is not merely a marker of differentiation status, but that SIRPγ itself plays an active role in modulating CD8 T cell fate. Specifically, we demonstrate that interindividual variability in SIRPγ expression strongly correlates with the composition of the peripheral CD8 T cell pool. Individuals with abnormally low SIRPγ expression on CD8 T cells have an increased frequency of CD27^−^ CD45RO^+^ effector-like and CD27^−^ CD45RO^−^ terminally differentiated T cells—phenotypes corresponding to more differentiated effector and terminal states. This suggests that low SIRPγ expression may predispose individuals to accelerated or dysregulated T cell differentiation.

To directly test the functional role of SIRPγ in regulating differentiation, we knocked down *SIRPG* in purified naïve CD8 T cells and examined their differentiation in response to low-dose TCR stimulation—a condition mimicking weak or suboptimal antigenic exposure. Knockdown of SIRPG resulted in a robust shift toward an effector-like CD45RO^+^ CD27^low^ phenotype, even in the absence of strong costimulation. This phenotype was consistent across multiple donors and underscores a previously unrecognized role for SIRPγ in enforcing a threshold for differentiation under suboptimal stimulatory conditions. These findings suggest that SIRPγ functions as a molecular brake, enforcing a threshold that prevents premature effector differentiation under limited activation conditions.

These findings place SIRPγ among a growing number of molecules that tune effector differentiation through modulation of activation thresholds. Similar to CD5 and NR4A family members, which act as negative regulators of TCR signal strength and prevent hyperactivation,^24^^,^^25^ SIRPγ may function to prevent low-affinity or subthreshold TCR signals that would otherwise lead to inappropriate effector programming. In the context of chronic infection or autoimmunity, such a regulatory mechanism would be crucial to maintain immune homeostasis.

Importantly, the CD45RO^+^ effector-like cells emerging from *SIRPG* KD cultures exhibited hallmark features of functional effector T cells. They displayed enhanced production of TNF-α, IFN-γ, and granzyme B upon suboptimal stimulation, and showed upregulation of the transcription factor T-bet—a key regulator of type 1 effector CD8 T cell differentiation.^26^^,^^27^ Notably, T-bet expression was restricted to the CD45RO^+^ subset, suggesting that these cells had undergone true effector programming. Thus, SIRPγ-deficient CD8 T cells not only acquire phenotypic markers of differentiation, but also gain functional effector capabilities.

Interestingly, the effector-like phenotype induced by SIRPγ KD could not be replicated by blocking its known ligand, CD47. Although SIRPγ-CD47 interaction has been proposed to mediate T cell migration,^28^ CD47 blockade failed to induce CD45RO expression or effector differentiation under suboptimal stimulation. This finding suggests that the regulatory function of SIRPγ in T cells is not solely mediated through CD47-dependent interactions. Our results reveal a CD47-independent role for SIRPγ in intracellular signaling that influences fate decisions. It is possible that SIRPγ may modulate signaling by associating with adaptor proteins or by influencing the spatial organization of signaling complexes at the immunological synapse. These possibilities warrant further investigation.

The interindividual variability in SIRPγ expression, particularly the presence of a naturally occurring SIRPγ^low^ subset, raises important questions about susceptibility to immune dysregulation. It is plausible that SIRPγ^low^ individuals may be predisposed to heightened effector responses, with potential implications for vaccine responsiveness, immunopathology, or even autoimmunity. In this context, we have shown that both relapsing-remitting multiple sclerosis and type 1 diabetes patients have significantly increased frequencies of SIRPγ^low^ CD8 T cells.

Taken together, our findings define a new role for SIRPγ in human CD8 T cell biology: it acts as a differentiation checkpoint that restricts effector programming under weak stimulatory conditions. This function may be critical for maintaining T cell homeostasis and preventing unnecessary immune activation. Interindividual differences in SIRPγ expression could therefore have important consequences for susceptibility to immune exhaustion, responsiveness to chronic infections, or propensity for autoimmunity. Notably, our previous work established that SIRPγ^low^ CD8 T cells are enriched in autoimmune conditions including type 1 diabetes and relapsing-remitting multiple sclerosis. Coupled with our current findings, this suggests that dysregulated SIRPγ expression may predispose to inappropriate effector T cell differentiation, contributing to the immune pathology observed in these diseases. Moreover, with growing interest in targeting SIRP family members for immunotherapy—particularly in the context of cancer^29^—our data suggest that modulating SIRPγ may represent a novel strategy to fine-tune T cell differentiation and function. Further exploration of the molecular mechanisms and regulatory networks involving SIRPγ will be essential to assess its potential as a therapeutic target in human immunopathology.

## Supplementary Material

vlag027_Supplementary_Data

## Data Availability

All data supporting the conclusion of this article are available from the corresponding author upon reasonable request.
