# Supplementary material for: SIRPγ limits effector differentiation of human CD8 T cells in response to subthreshold TCR-signaling
Source: Immunohorizons. 2026 Jun 21;10(6):vlag027. doi: 10.1093/immhor/vlag027 (PMC13283423; doi:10.1093/immhor/vlag027)
Supplement: vlag027_Supplementary_Data [file vlag027_supplementary_data.zip › Suppl Fig 1& 2.pdf_deleted]

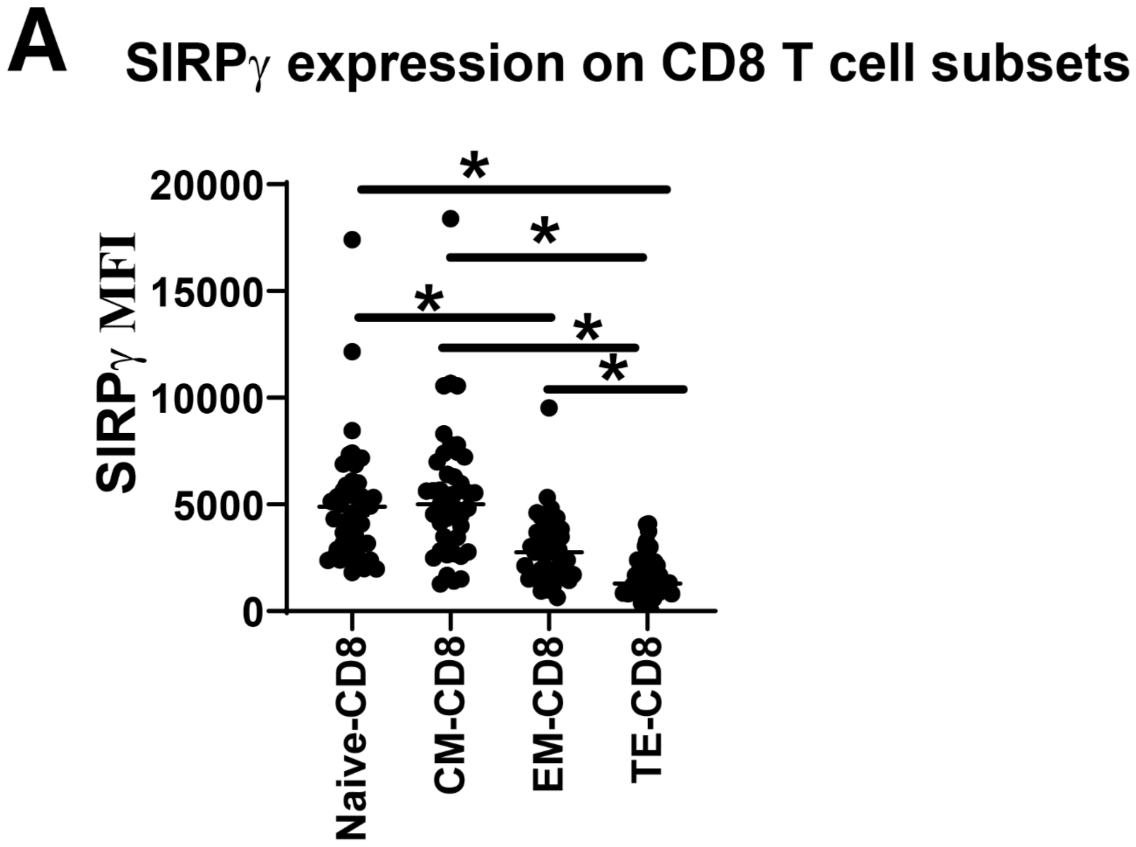

Supplementary Figure 1. SIRPγ expression is significantly reduced on effector memory and terminal effector CD8 T cells. Peripheral blood mononuclear cells (PBMCs) from SIRPγ^{high} donors were analyzed by flow cytometry to quantify SIRPγ expression across CD8 T cell differentiation subsets. SIRPγ expression is shown as mean fluorescence intensity (MFI). Each dot represents an individual donor. SIRPγ expression varied across differentiation states, with higher expression observed in naïve and CM CD8 T cells. SIRPγ expression is significantly reduced in EM and TE CD8 T cells. Data was compared using two-way ANOVA with Tukey's post hoc analysis and $p<0.05$ was considered significant.

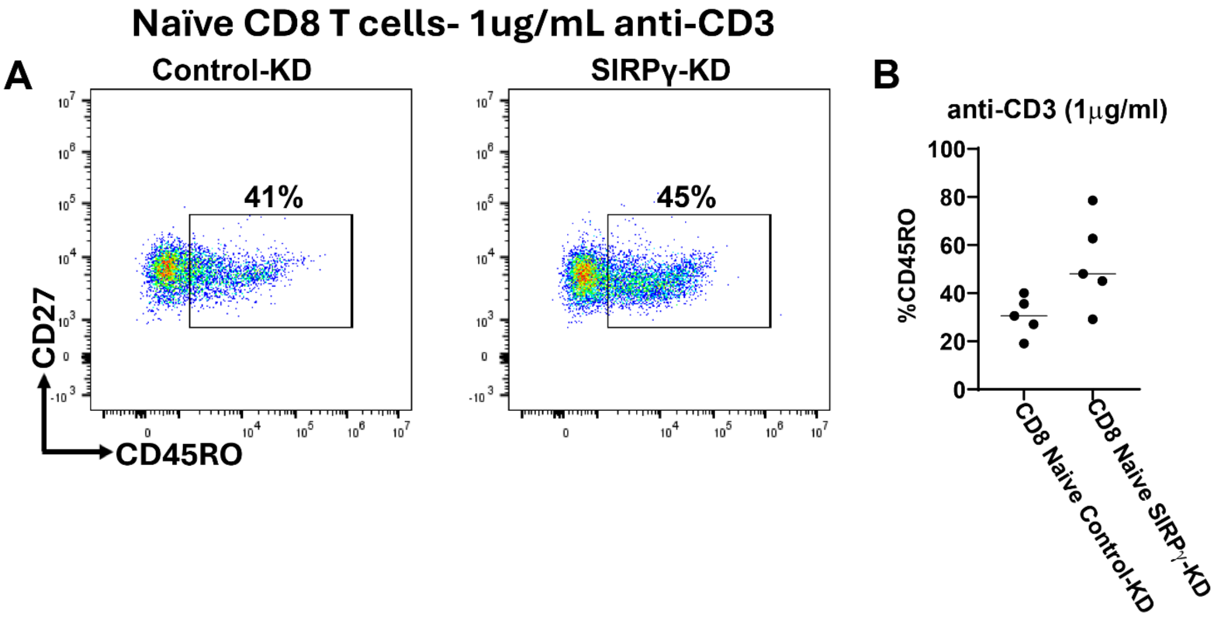

Supplementary Figure 2. Comparable effector like differentiation in C and SIRPγ -KD naïve CD8 T cells following optimal stimulation. (A) Representative flow cytometry dot plot showing CD27 and CD45RO expression in naïve CD8 T cells transfected with either control siRNA or SIRPG-specific siRNA following optimal anti-CD3 stimulation. **(B)** Although there is a trend toward an increase in effector-like differentiation upon SIRPγ-KD, however the difference was not significant. Statistical analysis was done using a paired t-test and p<05 was considered significant.
